# Isolated Adrenocorticotropic Hormone Deficiency Secondary to Chronic Opiate Use

**DOI:** 10.7759/cureus.9270

**Published:** 2020-07-19

**Authors:** Rishi Raj, Aasems Jacob, Ghada Elshimy, Jackson Smith

**Affiliations:** 1 Endocrinology, University of Kentucky, Lexington, USA; 2 Hematology and Oncology, University of Kentucky, Lexington, USA; 3 Endocrinology, Diabetes, and Metabolism, University of Arizona College of Medicine - Phoenix, Phoenix, USA; 4 Endocrinology, Diabetes, and Metabolism, University of Kentucky, Lexington, USA

**Keywords:** acth deficiency, secondary adrenal insufficiency, pituitary hormones, acth, pituitary disorders, opiate, chronic pain, chronic pain management

## Abstract

Although opiate use can result in various endocrine disorders, isolated adrenocorticotropic hormone (ACTH) deficiency resulting in secondary adrenal insufficiency remains uncommon. We present a case of a 54-year-old woman with a history of chronic opiate use who presented with a four-month history of worsening fatigue and syncopal episodes. Laboratory workup revealed a low ACTH with low baseline cortisol and normal levels of rest of the anterior pituitary hormones. The imaging study did not reveal any pituitary abnormality. The patient was diagnosed with opiate-induced isolated ACTH deficiency. Her symptoms improved after treatment with hydrocortisone. This case would further improve clinician's awareness towards opiate-induced endocrinopathies, including isolated ACTH deficiency, which can present with nonspecific signs and symptoms, creating a diagnostic challenge.

## Introduction

Opiate use has become a global epidemic in recent times. Although its use has been well recognized to cause various endocrinopathies, the mechanism of opiate-induced endocrinopathies remains poorly understood [1]. Opiated-induced hypogonadism is the most common endocrinopathy, with prevalence ranging from 35% to 90% among males [2]. Suppression of the hypothalamic-pituitary-adrenal (HPA) axis can also be seen. Due to its vague symptoms and latent presentation, opiate-induced secondary adrenal insufficiency is significantly underdiagnosed. This can lead to patient presentation in adrenal crisis, which carries very high morbidity and mortality. Discontinuation of opiate use can result in possible recovery of the HPA function. Appropriate hormone replacement with steroids remains the cornerstone of management. 

## Case presentation

A 54-year-old woman presented for evaluation of intermittent syncopal episodes for the past four months. She described syncopal episodes preceded with profuse sweating and shaking, which improved after meals. She also had fatigue, dizziness, and salt craving but denied any headache or weight loss. Her past medical history was significant chronic back pain treated with as-needed oral hydrocodone 5 mg every six to eight hours for one year. She was not on any other medications including NSAIDs. She was previously treated with acetaminophen without control for back pain and hence was treated with opiates. She did not have a prior history of head trauma, radiation, or pituitary/brain surgery. Family history was negative for autoimmune disease. She was a current smoker (10 packs per year). On physical examination, she was normotensive 136/83 mmHg with a pulse rate of 84 beats per minute, and a temperature of 97.6 degree Fahrenheit. Orthostatic vital signs were negative. She did not have hyperpigmentation of her skin. Visual field testing was normal. Cardiovascular and neurological causes of syncope were ruled out. Adrenocorticotropic hormone (ACTH) stimulation test with 250 mcg of intramuscular cosyntropin showed serum cortisol levels of <2.0, 7.3, and 8.3 ug/dL at time 0, 30, and 60 minutes respectively. Baseline ACTH level was 3.4 pg/mL (reference range: 7.2-63.3 pg/mL). The remaining laboratory workup is shown in Table 1. She had normal plasma renin activity with normal aldosterone, sodium, and potassium level. Estradiol level was in the postmenopausal range. MRI of the pituitary with and without contrast did not show a pituitary tumor or other abnormalities. She was diagnosed with opiate-induced isolated ACTH deficiency and was treated with physiologic dose of oral hydrocortisone 10 mg in the morning and 5 mg at 4 PM with significant improvement in her symptoms. She was reluctant to taper off the opioid as her pain was not controlled on other analgesics. At three months, she remained asymptomatic on the current dose of oral hydrocortisone.

**Table 1:** Summary of the laboratory workup. TSH, thyroid stimulating hormone; FT4, free thyroxine; IGF-1, insulin-like growth factor-1; FSH, follicle stimulating hormone; LH, luteinizing hormone

Laboratory test	Results	Reference ranges
Serum sodium	143 mmol/L	136-145 mmol/L
Serum potassium	3.8 mmol/L	3.7-4.8 mmol/L
Serum bicarbonate	23 mmol/L	22-29 mmol/L
Serum chloride	107 mmol/L	97-107 mmol/L
Serum calcium	9.7 mg/dL	8.9-10.2 mg/dL
Plasma aldosterone	3.1 ng/dL	0-30 ng/dL
Plasma renin activity	1.74 ng/ml/h	0.25-5.82 ng/ml/h
TSH	0.45 uIU/mL	0.4-4.2 uIU
FT4	1.5 ng/dL	0.8-1.8 ng/dL
IGF-1	118 ng/mL	53-287 ng/mL
Prolactin	5.9 ng/mL	4.4-23.3 ng/mL
FSH	38.1 mIU/ml	25.8-134.8 mIU/mL
LH	18.3 mIU/mL	7.7-58.5 mIU/mL
Estradiol	22 pg/mL	0-30 pg/mL (postmenopausal)

## Discussion

The first case of isolated ACTH deficiency was described in 1954 by Steinberg et al. [3]. Among the pediatric population, it is exclusively due to genetic mutations. However, in recent times, opiate use has emerged as one of the leading causes of secondary adrenal insufficiency. Nonetheless, it is believed to be significantly underdiagnosed. Besides opiate use, other conditions that can lead to isolated ACTH deficiency include autoimmune conditions, lymphocytic neurohypophysitis, and immune checkpoint inhibitors [4-8]. Despite the fact that it has been decades since the first reported case, this remains a poorly understood entity. From limited research, opioids are known to work on the hypothalamic-pituitary-adrenal (HPA) axis at three primary opioid receptors (μ, δ, and κ), leading to a lack of ACTH secretory response to corticotropin-releasing hormone (CRH) [9].

The true prevalence of opiate-induced secondary adrenal insufficiency remains unclear and currently available data are primarily from few retrospective studies and case reports [10]. Few studies have suggested that chronic use of opiates can disrupt the normal circadian rhythm of cortisol secretion as well [11].

The clinical presentation of isolated ACTH deficiency is nonspecific and can include fatigue, anorexia, weakness, hypoglycemia, and loss of consciousness [12-13]. Our patient presented with fatigue, dizziness, salt craving, and intermittent loss of consciousness, which could be from hypoglycemia after prolonged fasting. Unfortunately, our patient did not have documentation of low glucose levels and we can only assume her symptoms to be due to hypoglycemia which resolved after treatment of secondary adrenal insufficiency. Apart from vague presenting symptoms, these patients also have a latent presentation and very few patients seek timely medical attention, which can significantly delay the diagnosis. Rarely, these patients can present in adrenal crisis with hypovolemia, hyponatremia, and hypoglycemia after an acute illness [13].

Once isolated ACTH deficiency is suspected, the diagnosis can be confirmed by demonstrating selective functional loss of corticotropes, evidenced by low levels of ACTH and cortisol along with normal levels of the other five anterior pituitary hormones - thyroid stimulating hormone (TSH), follicle stimulating hormone (FSH), luteinizing hormone (LH), insulin-like growth factor 1 (IGF1), and prolactin [14-16]. Some studies have also suggested a demonstration of the failure of adrenal glands to respond to CRH along with normal secretory indices of other anterior pituitary hormones to establish the diagnosis of isolated ACTH deficiency [13]. The CRH stimulation test can further help in distinguishing between supra-pituitary and pituitary dysfunction as the underlying etiology of isolated ACTH deficiency, which in turn could be from CRH deficiency, CRH resistance, or dysfunction of pituitary corticotropes. Although a combination of CRH stimulation test and insulin tolerance test can be used to diagnose the underlying etiology of isolated ACTH deficiency, most patients with isolated ACTH deficiency show a pituitary defect following the CRH stimulation test, suggesting an extremely low incidence of the supra-pituitary defect [17]. Besides, insulin tolerance test (ITT) is not used nowadays due to its complications.

Nonetheless, isolated ACTH deficiency is treated similar to any case of secondary adrenal insufficiency with physiologic doses of hydrocortisone given in divided doses with recommendations to increase the doses at times of stress such as fever, infection, or other illnesses. Besides, steroids, limited studies have shown a possible role of intranasal ACTH replacement therapy in patients with isolated ACTH deficiency [18].

## Conclusions

Opioid-induced isolated ACTH deficiency is an underdiagnosed condition in clinical practice, which can be attributed to under-reporting of symptoms by patients as well as poor clinician awareness. Healthcare professionals should be familiar with the adverse events associated with opiates. Furthermore, they should caution patients at the initiation of treatment, particularly if long-term treatment with opiates is desired. Additionally, patients on chronic opiates should be appropriately monitored with hormonal workup. 
